# Spectroscopic Methods in the Evaluation of Modified Vegetable Base Oils from *Crambe abyssinica*

**DOI:** 10.3390/molecules23123243

**Published:** 2018-12-07

**Authors:** Michał Szmatoła, Justyna Chrobak, Rafał Grabowski, Jolanta Iłowska, Julia Woch, Iwona Szwach, Izabela Semeniuk, Jolanta Drabik, Małgorzata Wrona, Rafał Kozdrach, Beata Orlińska, Mirosława Grymel

**Affiliations:** 1 Institute of Heavy Organic Synthesis “Blachownia”, Energetykow 9, 47-225 Kedzierzyn-Kozle, Poland; chrobak.j@icso.com.pl (J.C.); grabowski.r@icso.com.pl (R.G.); ilowska.j@icso.com.pl (J.I.); woch.j@icso.com.pl (J.W.); szwach.i@icso.com.pl (I.S.); semeniuk.i@icso.com.pl (I.S.); 2 The Institute for Sustainable Technologies–National Research Institute, Kazimierza Pulaskiego 6/10, 26-600 Radom, Poland; jolanta.drabik@itee.radom.pl (J.D.); malgorzata.wrona@itee.radom.pl (M.W.); rafal.kozdrach@itee.radom.pl (R.K.); 3 Department of Organic Chemical Technology and Petrochemistry, Silesian University of Technology, B. Krzywoustego 4, 44-100 Gliwice, Poland; beata.orlinska@polsl.pl; 4 Department of Organic Chemistry, Bioorganic Chemistry and Biotechnology, Silesian University of Technology, B. Krzywoustego 4, 44-100 Gliwice, Poland; miroslawa.grymel@polsl.pl; 5 Biotechnology Center of Silesian University of Technology, B. Krzywoustego 8, 44-100 Gliwice, Poland

**Keywords:** vegetable oil, *Crambe abyssinica* oil, chemical modification, oxidation, *N*-hydroxyphthalimide, Raman spectroscopy

## Abstract

Raw vegetable oil from *Crambe abyssinica* was subjected to oxidative treatment to enhance its viscosity. The oxidation processes were carried out in the presence of *N*-hydroxyphthalimide with or without supercritical CO_2_ as a solvent. Four spectroscopic techniques (Raman, UV-VIS, FT-IR, NMR) were applied to assess the chemical changes taking place during the oxidation. Raman and NMR spectroscopy proved best in the assessment of the chemical transformations leading to increased viscosity of the modified vegetable oil.

## 1. Introduction

Oils are a group of products which are characterized by a high variability of their chemical and physical properties. The chemical analysis of vegetable oils is arduous because they consist of complex mixtures of chemical compounds and the evaluation of the results can also be disturbed by a matrix effect [1]. However, characterization of these oils can be performed using a great variety of analytical methods and chemometric methodologies. Different techniques for fats and oils analysis have been developed so far and a large part of them is based on liquid or gas chromatography [2,3]. 

There has been a growing demand to replace traditional analytical methods with instrumental methods. The superiority of the latter is due to their high sensitivity, low limits of detection, speed of analysis and automated operation. Modern instrumental techniques include spectroscopic methods that are successfully used in qualitative and quantitative analysis in various industries, including the chemical and food industries, electronics or metallurgy. Furthermore, spectroscopic methods can be used in conjunction with advanced separation methods, which apart from comprehensive qualitative and quantitative analysis of isolated intermediates, enable the direct analysis of final products. Numerous scientific publications prove the usefulness of spectroscopic methods in the study of the properties of vegetable oils and, above all, the assessment of the quality of edible oils [4,5]. The analysis of vegetable oils can be carried out with the use of absorption spectroscopy in the area of the ultraviolet-visible spectral region (UV-Vis) [6,7,8], infrared spectroscopy (IR) [9,10,11,12,13,14], nuclear magnetic resonance spectroscopy (NMR) [15,16,17] and Raman spectroscopy [18,19,20,21].

The performance of base oils mostly depends on their physiochemical properties. The characteristics of base oils play a key role in the ultimate formulation of high-quality lubricants. Several publications concern the use of instrumental methods to determine the properties of various base oils [22,23,24,25,26] and lubricants [27,28,29,30,31,32]. However, these articles relate mainly to the analysis of mineral or synthetic base oils. Lubricants based on vegetable oils seem to be an attractive alternative to conventional lubricants. Structurally, vegetable oils are mixtures of natural fatty acid esters and are an alternative to replace the commonly used mineral oils due to their natural occurrence and their fast biodegradation rates which could reduce environmental risks.

The base oils obtained in the innovative process of vegetable oils modification, presented in our previous article [33], were subjected to various tests to determine the changes that have occurred in the chemical composition. The aim of this paper is to demonstrate the applicability of spectroscopic techniques, including UV-VIS spectroscopy, NMR, infrared and Raman spectroscopy, as the methods of analysis to evaluate the result of the modification reactions and determine their effect on the structural changes between unmodified vegetable oils and oil bases obtained as a result of oxidation reactions. 

## 2. Results and Discussion

### 2.1. Oxidative Modification of Raw Crambe Oil

The raw vegetable oil from *Crambe abyssinica* (A) is characterized by a high content of both unsaturated erucic acid and natural antioxidants. *Crambe abyssinica* belongs to the cabbage family (*Brassicaceae*). It is an oilseed crop which originated from the Mediterranean region. It has also been grown in other regions e.g., tropical and subtropical parts of Africa, Central and West Asia, Europe, North and South America [34]. The oil is used as a lubricant, corrosion inhibitor and in the production of synthetic rubbers, nylons or plastic films [35]. The raw oil was modified to obtain higher viscosity class (VG 150) oils via an oxidative treatment. The oil, catalyst, radical initiator and solvent were vigorously stirred at elevated temperature and pressure under an oxygen atmosphere. The process was stopped after a specified amount of oxygen had been absorbed. *N*-Hydroxyphthtalimide (NHPI) was used as a catalyst, azobisisobutyronitrile (AIBN) as a radical initiator and supercritical carbon dioxide (scCO_2_) as a solvent. Reactions without scCO_2_ were performed for comparison. The modified oils were marked as A_CO_2_ and A_O_2_ accordingly. After the experiments all modified oils were tested to determine their kinematic viscosity, peroxide number (LN), iodine number (LJ), saponification number (LZ) and acid number (LK). The results are summarized in Table 1. In this Table, mean values of the measurements (three repetitions) and the values of standard deviations are presented.

### 2.2. Oxidative Stability Tests of the Modified Oils

The raw vegetable oil (A) with VG 46 viscosity class and VG 150 (A_CO_2_ and A_O_2_) oils obtained through modification were subjected to Rapid Small Scale Oxidation Test (RSSOT, PetroOxy) to evaluate their oxidation stability. This method consists in the acceleration of the oxidation process for a chosen sample at an elevated temperature and exposure to the excess of oxygen. The sample is placed in a small chamber and is exposed to the oxygen under pressure at a certain temperature. The temperature is kept constant and the pressure is constantly measured until a specified pressure drop is detected. During our research tests were performed in isothermal conditions, at 80 °C and 120 °C. Measurements were taken until pressure dropped by 10%. The time of oxidation induction was determined on that basis (Table 2). In this Table, mean values of the measurements (three repetitions) and the values of standard deviations are presented.

### 2.3. Spectroscopic Methods

The obtained oils were analyzed using spectroscopic techniques. For the following materials, Raman, UV-VIS, IR and NMR spectra were recorded: raw vegetable oil (A) with VG 46 viscosity class before and after the PetroOxy test (A_80, A_120 for samples tested at 80 °C and 120 °C respectively); modified oils (A_O_2_ and A_CO_2_) with VG 150 viscosity class before and after the PetroOxy test (A_O_2__80 and A_O_2__120; A_CO_2__80 and A_CO_2__120, for samples tested at 80 °C and 120 °C respectively). 

#### 2.3.1. Raman Spectroscopy

The raw vegetable oil from *Crambe abyssinica* (A) is composed mainly of unsaturated fatty acids with the following molecular structure: erucic acid (C22:1, 63.77%), oleic acid (C18:1, 15.07%), linoleic acid (C18:2, 13.16%) and linolenic acid (C18:3) [36,37]. Unsaturation is the main cause of the reactivity of the oil towards oxygen.

**Figure 1:**
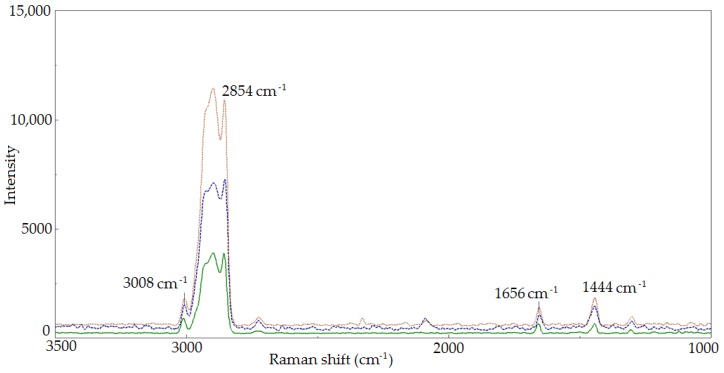
Raman spectra of A oil (green line) and A_CO_2_ (blue line) as well as A_O_2_ (red line) modified oils, in the 3500 cm^−1^–1000 cm^−1^ range.

Figure 1 shows the Raman spectra of oils obtained in the modification process (A_CO_2_ and A_O_2_) in comparison with the raw oil (A). The use of Raman spectroscopy allowed us to evaluate the influence of the modification process and thermal activation on changes in structure of the researched oils. The band assignments of the Raman spectra of initial A oil and modified A_CO_2_ and A_O_2_ oils are reported in Table 3 [38,39,40,41,42,43].

**Figure 2:**
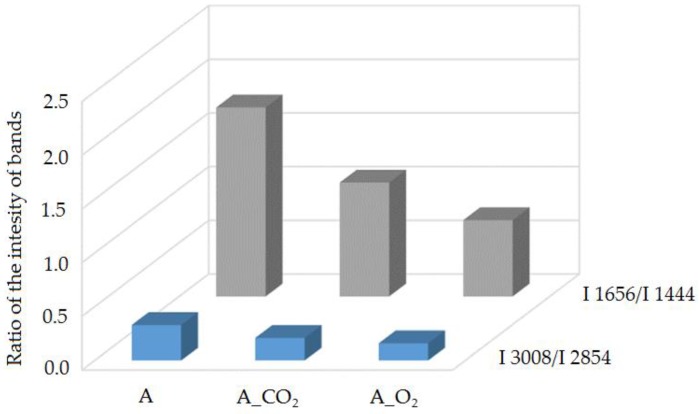
The influence of the modification process on the change in the unsaturation degree of fatty acids in the researched oils: A, A_CO_2_ and A_O_2_, expressed by I_1656_/I_1444_ and I_3008_/I_2854_ bands intensity ratio.

Based on the Raman spectra analysis of the studied A, A_CO_2_ and A_O_2_ oils, the modification influence on the fatty acids unsaturation degree change was evaluated. To do this, the ratio of bands intensity at 1656 and 1444 cm^−1^ (I_1656_/I_1444_) as well as bands at 3008 and 2854 cm^−1^ (I_3008_/I_2854_), resulting from the vibration of characteristic functional groups, was calculated. The ratio of the number of C=C double bonds to the number of C-C single bonds in the fatty acids of the evaluated oils was determined, and thus the influence of the modification process conditions on unsaturation degree change was estimated (Figure 2).

**Figure 3:**
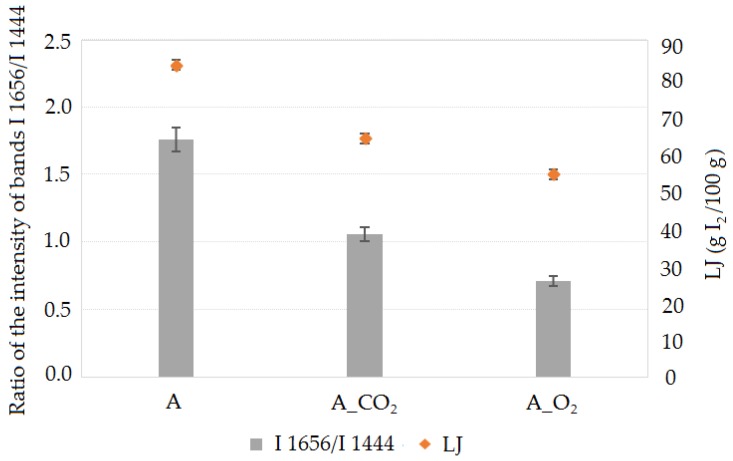
The influence of the A oil modification process on the fatty acids unsaturation change in A_CO_2_ and A_O_2_ oils, expressed by the I_1656_/I_1444_ bands intensity ratio and LJ iodine number.

The modification process of oil A, conducted for the purpose of obtaining base oils with a higher viscosity class, led to changes in the structure. It was found that the oil modification process carried out in the presence of CO_2_ solvent was more beneficial because the established viscosity of A_CO_2_ oil was obtained by smaller structure changes, evaluated on the basis of the I_1656_/I_1444_ and I_3008_/I_2854_ band intensity ratio. The basis for comparison with analytical parameters, especially iodine number LJ, was identified bands from the Raman spectra of the studied I_1656_/I_1444_ oils, which characterized the unsaturation degree of the fatty acids (Figure 3).

It was possible to compare the results of Raman spectroscopy with the iodine number because in both cases the number of double bonds in oil is determined. Raman spectroscopy allowed us to calculate the ratio of the number of C=C double bonds to the number of C-C single bonds in the oil, where a higher value of the ratio of those bands indicates a higher degree of oil unsaturation. The iodine number determines the number of grams of iodine necessary for the saturation of multiple bonds. The higher the iodine number value the greater the number of double bonds that the fatty acids in the oil contain. It was indicated that the Raman spectra allowed us to estimate the iodine number of oils.

**Figure 4:**
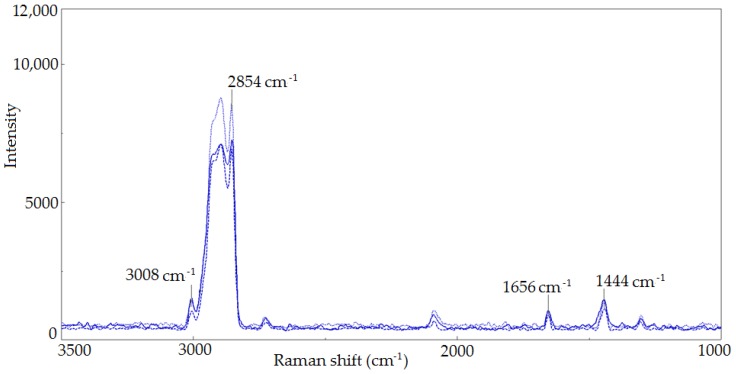
Raman spectra of A_CO_2_ oil (blue line), A_CO_2__80 oil, oxidized at 80 °C (----- blue line) and A_CO_2__120 oil, oxidized at 120 °C (●●●●● blue line), in the 3500 cm^−1^–1000 cm^−1^ range.

**Figure 5:**
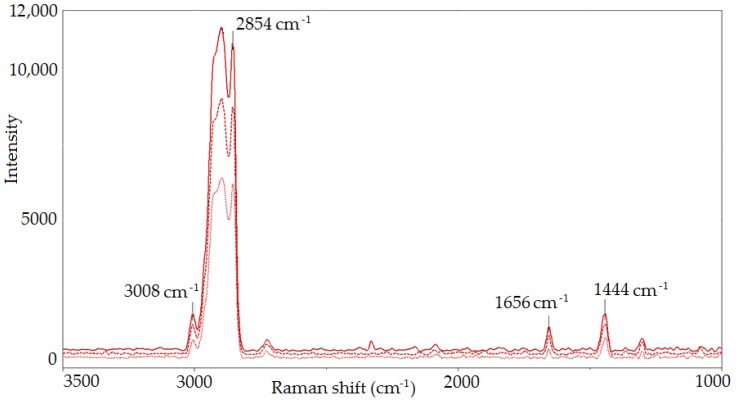
Raman spectra of A_O_2_ oil (red line), A_O_2__80 oil, oxidized at 80 °C (----- red line) and A_O_2__120 oil, oxidized at 120 °C (●●●●● red line), in the 3500 cm^−1^–1000 cm^−1^ range.

The use of Raman spectroscopy also allowed the evaluation of the thermal activation’s influence on the unsaturation degree change of the researched oils. Samples of A, A_CO_2_ and A_O_2_ oils were subjects to thermal activation in a laboratory test under the conditions of continuous oxygen activity at 80 °C and 120 °C. Based on that, the oils’ oxidation stability was evaluated as well as the oxidation induction time (Table 1). During the tests the Raman spectra of the oxidized oils were recorded. They were compared with the spectra obtained before the oxidation process. Exemplary spectra are shown below in Figure 4 and Figure 5. 

**Figure 6:**
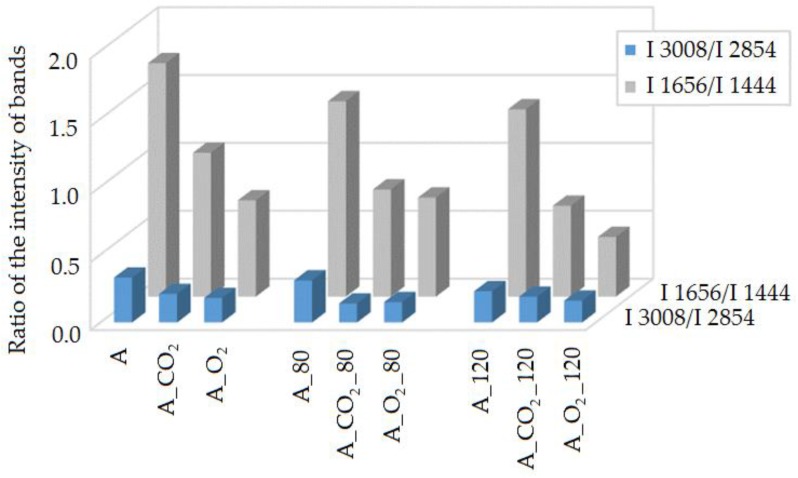
The influence of oxidation on the change in fatty acids unsaturation degree of the researched oils before and after oxidation, at 80 °C and 120 °C, expressed by I_1656_/I_1444_ and I_3008_/I_2854_ band intensity ratio.

The degree of unsaturation of the oxidized oils and the influence of oxidation on that change were evaluated on the basis of the obtained spectra. The band intensity ratio at 1656 and 1444 cm^−1^ (I_1655_/I_1438_) as well as at 3008 and 2854 cm^−1^ (I_3008_/I_2854_), resulting from the vibration of characteristic functional groups, was determined. Thus, the influence of the modification and oxidation process conditions on the unsaturation degree change was estimated. The band intensity ratio determined for individual oils before and after oxidation was compared—carried out at 80 °C and 120 °C for A oil before and A_80, A_120 after oxidation, A_CO_2_ oil before and A_CO_2__80 A_CO_2__120 after oxidation, and for A_O_2_ oil before and A_O_2__80 A_O_2__120 after oxidation (Figure 6).

In the oils’ spectra after the oxidation test at 80 °C and 120 °C, bands at 2854 cm^−1^ and 1656 cm^−1^ were smaller than those for the initial oils. For all studied oils after the test, the change in structure was the smallest for initial A oil in comparison to modified A_CO_2_ and A_O_2_ oils.

The determined oxidation stability confirmed the higher resistance to oxidation of raw oil in comparison to modified oils, which is connected to the oil’s molecular structure. The oil modification process initiated changes in the structure and the thermal activities augmented the changes. Oils in the VG 150 viscosity class result from the modification, but the modification process carried out in the presence of CO_2_ had a smaller effect on the fatty acid unsaturation degree-diagnostic bands present in the Raman spectrum, which was also confirmed through analytical methods.

#### 2.3.2. UV-VIS Spectroscopy

**Figure 7:**
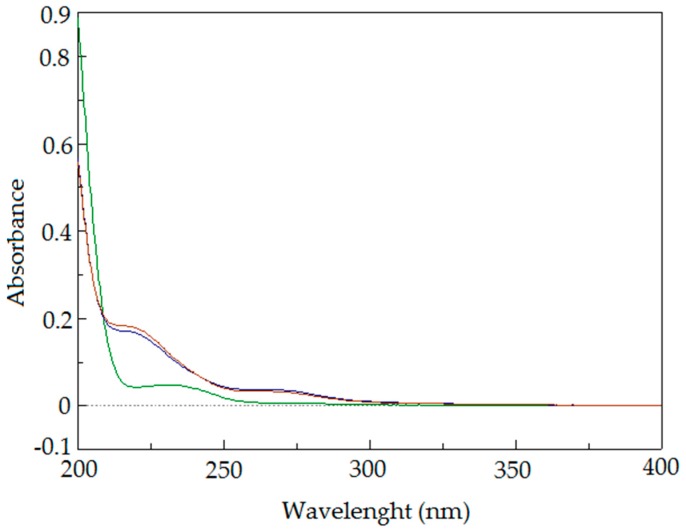
UV-VIS spectra of modified oils A_CO_2_ (blue line) and A_O_2_ (red line) superimposed on the spectrum of raw *Crambe abyssinica* oil A (green line).

The raw oil (A) does not exhibit any absorption above 300 nm. In the 200–400 nm region, a weak absorption band is present λ = 234 nm, ε = 0.046. In the spectra of both modified oils, two additional absorption maxima are present: λ = 218 nm, ε = 0.18 and λ = 268 nm, ε = 0.032 for A_O_2_ and λ = 218 nm, ε = 0.168 and λ = 268 nm, ε = 0.036 for A_CO_2_ (Figure 7).

#### 2.3.3. FT-IR Spectroscopy

**Figure 8:**
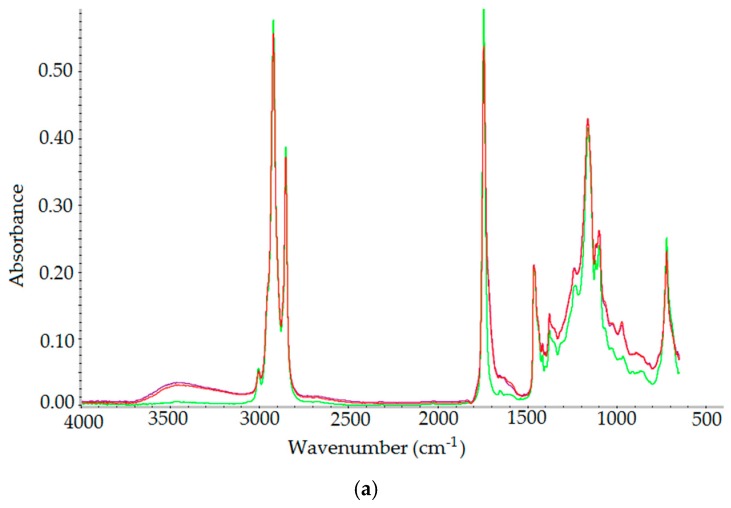

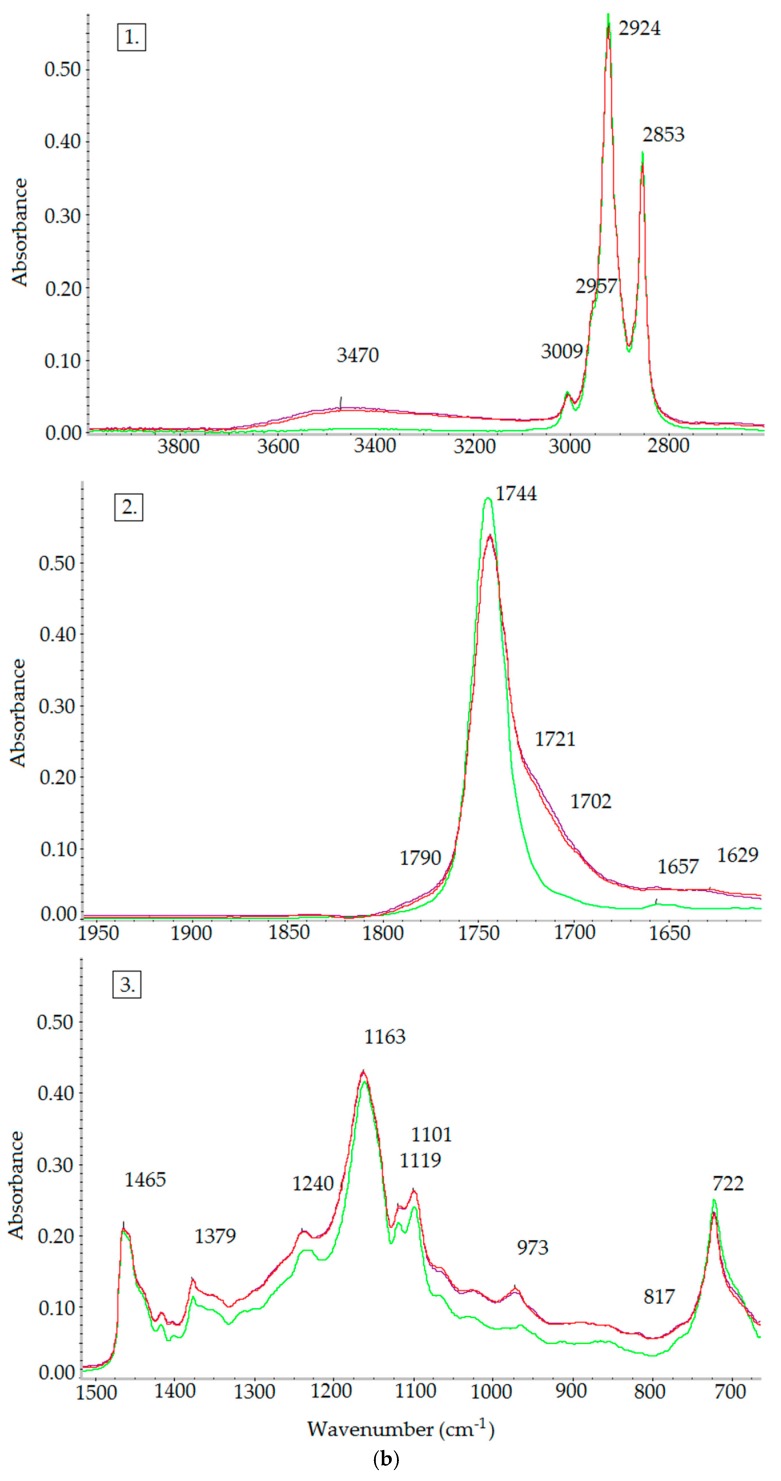
(**a**) ATR-FTIR spectra of oils: raw *Crambe abyssinica* oil A (green line), A_O_2_ (red line), A_CO_2_ (blue line). (**b**) ATR-FTIR spectra of oils: raw *Crambe abyssinica* oil A (green line), A_O_2_ (red line), A_CO_2_ (blue line): **b1.** In the region of 3800–2400 cm^−1^ **b2.** In the region of 1950–1600 cm^−1^ **b3.** In the region of 1500–650 cm^−1^.

Figure 8 shows the ATR-FTIR spectra of raw oil A and modified oils A_O_2_ and A_CO_2_. The major bands are associated with trifatty acid esters of glycerol which are main components of the oils. 

All recorded spectra showed absorption bands at different wavenumbers, as follows: 3005 cm^−1^ (γ_C-H_ of *cis* double bonds =CH), shoulder at 2955 cm^−1^ (γ_C-H_ of aliphatic CH_3_), 2922 and 2853 cm^−1^ (γ_C-H_ of CH_2_), 1744 cm^−1^ (γ_C=O_ of ester carbonyl functional groups of triglycerides (O-C=O), weak shoulder at 1719 and 1700 cm^−1^ (γ_C=O_ of carbonyl functional groups of ketones and free fatty acids, 1655 cm^−1^ (γ_C=C_ of *cis*-olefins), 1463, 1458 cm^−1^ (δ_C-H_ of CH_2_ and CH_3_ aliphatic groups), 1418 cm^−1^ (rocking vibration of C-H bonds of *cis*-disubstiuted olefins), 1397 cm^−1^ (δ_C-H in plane_ of C-H of *cis*-olefinic groups), 1376 cm^−1^ (δ_C-H_ of C-H of CH_2_ groups), 1237, 1160, 1118 and 1096 cm^−1^ (γ_C-O_ of ester groups), 970 cm^−1^ (δ_C=C out-of-plane_ of disubstituted olefins), 722 cm^−1^ (overlapping of aliphatic CH_2_ rocking vibration and out of plane vibration of *cis*-disubstituted olefins) [44]. 

By comparing the spectra of raw and modified oils in 3800–2400 cm^−1^ region, the change in the absorbance at 3465 cm^−1^ (γ_O-H_ in OH groups) is noticeable, as the height of this band increased after modification. The band at 3005 cm^−1^ decreased slightly after treatment and the increase in absorbance at 970 cm^−1^ indicated the formation of *trans* C=C bonds.

The examination of ATR-FTIR spectra of raw and modified oils revealed the changes in the following regions: the OH stretching region of hydroxyl groups between 3600 and 3100 cm^−1^, hydrogen stretching vibrations between 3050 and 2800 cm^−1^ and carbonyl stretching vibrations between 1800 and 1680 cm^−1^. These changes reflect the modification process leading to the alteration of structure and properties of the raw *C*. *abyssinica* oil.

Comparing the spectra of raw and modified oils, absorbance changes at 3465 cm^−1^ (γ_O-H_ in OH groups) are observed. The height of this band increased after modification most likely due to the formation of -COOH. Likewise the absorbance between 1800 and 1680 cm^−1^ after modification decreased and the broadening of the band is observed, which is associated with appearance of new carbonyl groups originated from ketones, aldehydes and free fatty acids. The resulting decrease of band at 1744 cm^−1^ is caused by the loss of the carbonyl groups of fatty esters. The band at 3005 cm^−1^ and 1650 cm^−1^ slightly decreased after treatment, which is associated with the decrease of *cis* C=C bonds. At the same time absorbance at 970 cm^−1^ increased indicating the formation of *trans* C=C bonds. 

**Figure 9:**
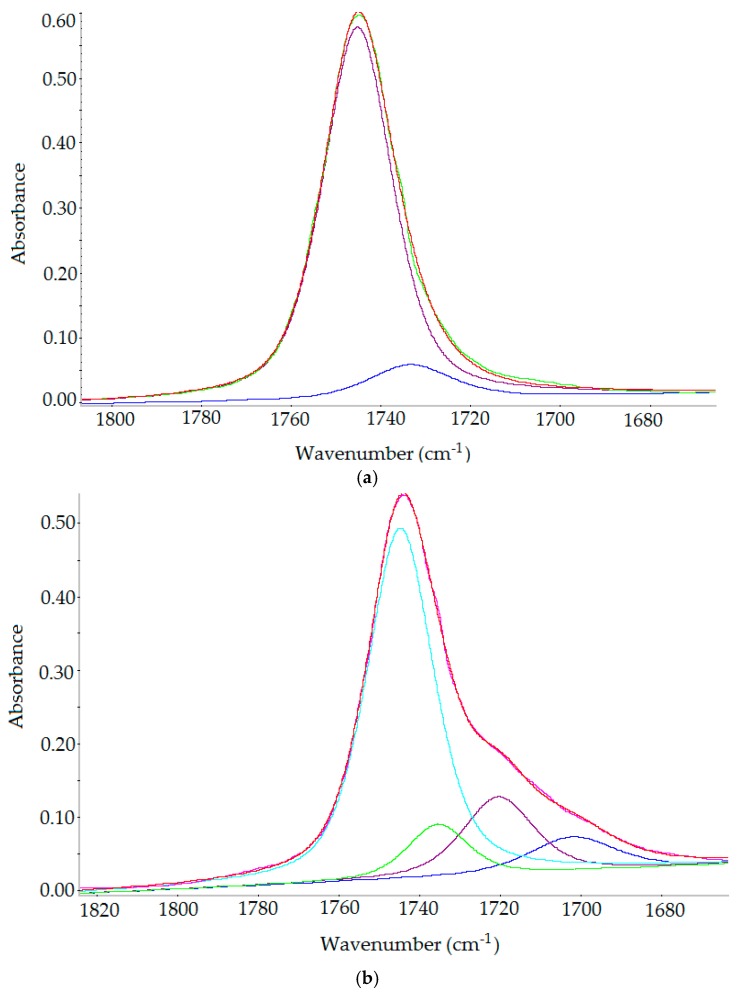

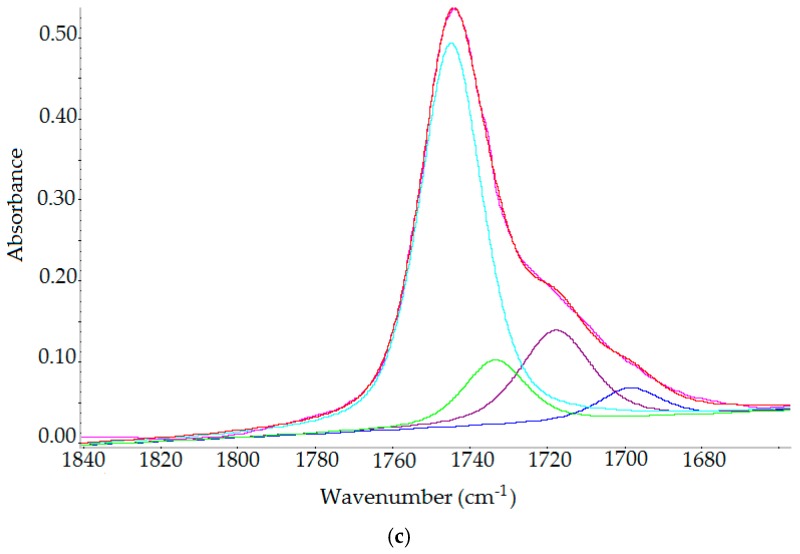
(**a**) Deconvolution of carbonyl bands of raw oil A: 1745 cm^−1^ (violet line), 1734 cm^−1^ (blue line), original spectrum (green line), total of all deconvolution bands (red line). (**b**) Deconvolution of carbonyl bands of oil A_O_2_: 1745 cm^−1^ (sky-blue line), 1735 cm^−1^ (green line), 1720 cm^−1^ (violet line), 1702 cm^−1^ (blue line), original spectrum (pink line), total of all deconvolution bands (red line). (**c**) Deconvolution of carbonyl bands of oil A_CO_2_: 1745 cm^−1^ (sky-blue line), 1734 cm^−1^ (green line), 1718 cm^−1^ (violet line), 1700 cm^−1^ (blue line), original spectrum (pink line), total of all deconvolution bands (red line).

Deconvolution of the spectra of raw oil A in the carbonyl range between 1770 and 1680 cm^−1^ reveals two carbonyl bands at 1745 cm^−1^ and at 1733 cm^−1^ while deconvolution of the modified oils A_O_2_ and A_CO_2_ spectra shows additional two carbonyl bands at about 1720 cm^−1^ and at 1700 cm^−1^ which in comparison does not show in case of raw oil (A) (Figure 9a–c). The band at 1720 cm^−1^ results from the creation of aldehydes, ketones, esters and band at 1700 cm^−1^ represents the appearance of free fatty acids [45]. Gaussian/Lorenzian deconvolution model with middle sensitivity and wave width of 4 cm^−1^ gave good compatibility of original spectrum and the total of all deconvolution bands. 

ATR-FTIR spectroscopy thus seems to be an appropriate and comprehensive tool for monitoring the chemical changes appearing during modifications of the *Crambe abissinica* oil.

#### 2.3.4. NMR Spectroscopy

**Figure 10:**
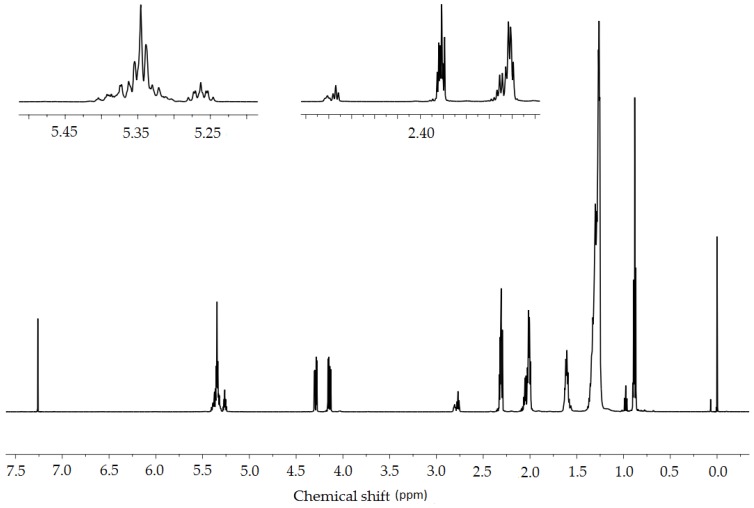
^1^H-NMR spectrum (600 MHz, CDCl_3_) of crude *Crambe abyssinica* oil: δ 0.88 (t, 9H, *J* = 0.88 Hz, CH_2_CH**_3_**), 1.35–1.20 (m, 70H, (CH_2_)_n_), 1.65–1.55 (m, 6H, CH_2_CH**_3_**), 2.10–1.97 (m, 12H, CH_2_-CH=CH-CH_2_-), 2.34–2.28 (m, 6H, CH_2_COO), 2.83–2.75 (m, 2H, CH=CH-CH_2_-CH=CH), 4.32–4.12 (m, 4H, OCH_2_CH(O)CH_2_O), 5.28–5.20 (m, 1H, OCH_2_CH(O)CH_2_O), 5.43–5.28 (m, 8H, CH=CH).

**Figure 11:**
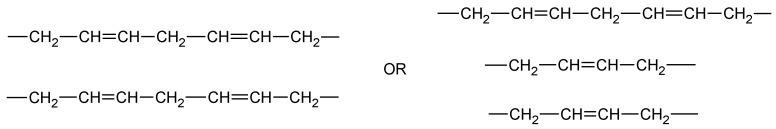
The double bond distribution possibilities.

Based on the ^1^H-NMR spectra of crude *Crambe abyssinica* oil (Figure 10), it was determined that signals at a chemical shift of 0.9 ppm originate from the -CH_3_ groups. The integral calculations (Table 4) were made by assuming that there are three such groups in the triglyceride molecule, equal to nine protons. On this basis, it was determined that the multiplet at 5.35 ppm corresponds to eight hydrogen atoms derived from vinyl groups (-CH=CH-). Therefore, the distribution of double bonds in the molecular chains of triglyceride is possible in two ways: two moieties consisting of two double bonds connected by a methylene group (the so called bis-allylic group =CH-CH**_2_**-CH=) or one such moiety and two isolated double bonds (Figure 11).

**Figure 12:**
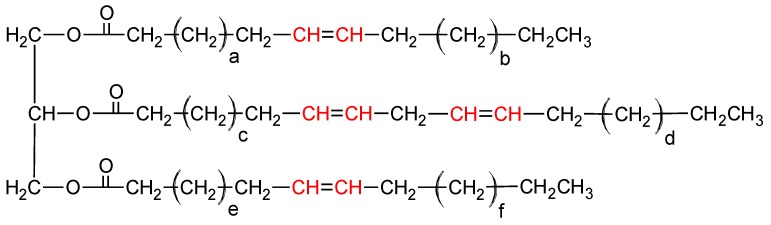
The general structure of the triglyceride.

Based on the integrals of peaks originating from the allylic methylene groups (2.0 and 2.8 ppm), it was determined that there are two hydrogen atoms originating from the bis-allylic methylene group according to the structure =CH-CH_2_-CH= and 12 hydrogen atoms that are derived from the allylic protons -CH_2_-CH=CH-CH_2_-. This information allowed us to figure out the general triglyceride structure eliminating one of the above possibilities. In only one chain are there two double bonds (Figure 12).

In addition, the number of hydrogen atoms forming the -CH_2_- chains was calculated (70 H) and this allowed us to estimate the triglyceride structure. The fatty acid chains that create the molecule probably originate from erucic acid 22:1 and linoleic acid 18:2. Therefore:

$$\begin{array}{c} a=7\\ b=7\\ c=5\\ d=2\\ e=7\\ f=7 \end{array}$$

**Figure 13:**
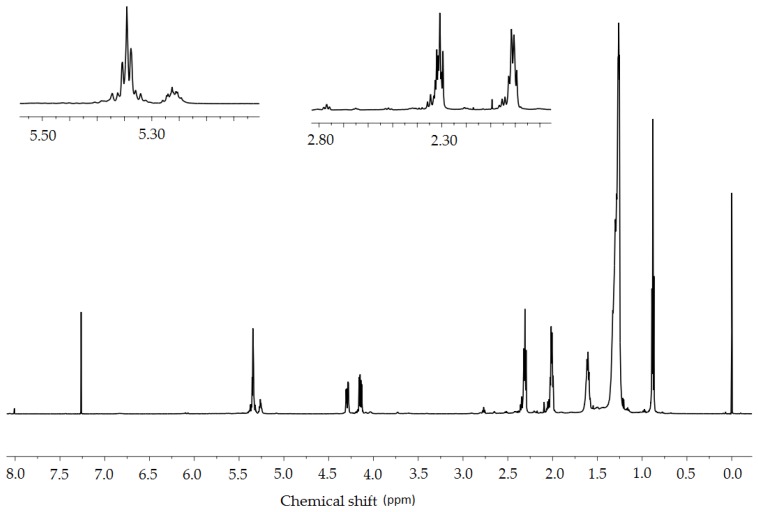
^1^H-NMR spectrum of modified *Crambe abyssinica* oil in the presence of oxygen.

**Figure 14:**
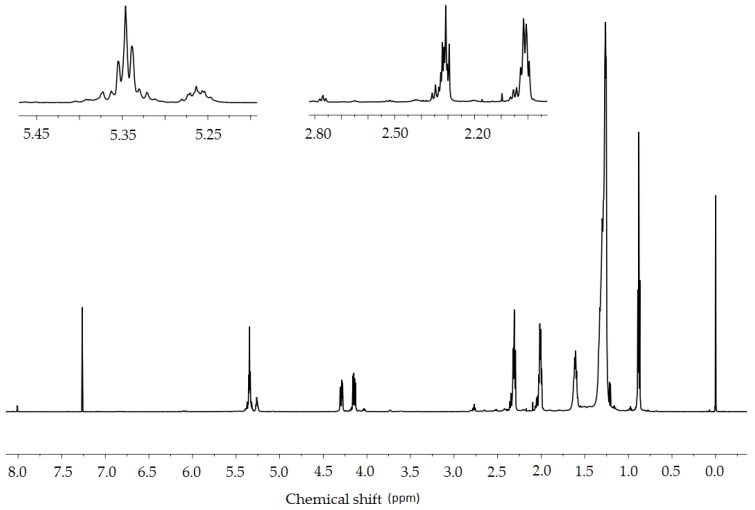
^1^H-NMR spectrum of modified *Crambe abyssinica* oil in the presence of oxygen and CO_2_.

The structural changes that occurred as a result of the oxidation of *Crambe abyssinica* oil were determined with the use of ^1^H-NMR spectra (Figure 13 and Figure 14). These changes are mainly demonstrated by the decreased intensity of three signals (2.0, 2.8 and 5.35 ppm). Comparing the spectrum of the modified oil with the crude oil, a decrease in the value of the integral of the signal deriving from the vinylic protons -CH=CH- is noticeable (5.35 ppm). Using these values (Table 4), it is possible to calculate the degree of conversion of double bonds:

(1) $$\alpha_{\mathrm{CH}=\mathrm{CH}}=\frac{5.54-3.42}{5.54}\times 100\%=38\%$$

Another issue worth considering is the signal with 2.8 ppm chemical shift coming from the hydrogen atoms between the two double bonds =CH-CH_2_-CH=. This signal also decreases considerably which indicates the disappearance of double bonds.

In addition, the signal with 2.0 ppm chemical shift derived from the hydrogen atoms in the CH_2_-CH=CH-CH_2_ structure is reduced which is another proof of a change in the structure confirming the efficiency of the oxidation of double bonds.

The analysis described above also applies to *Crambe abyssinica* oil oxidized in the presence of carbon dioxide. The differences between the spectra of modified oils are slight. Decreased intensity of signals from vinylic protons (5.35 ppm chemical shift) allowed us to calculate the degree of conversion of double bonds:

(2) $$\alpha_{\mathrm{CH}=\mathrm{CH}}=\frac{5.54-3.58}{5.54}\times 100\%=35\%$$

In the presence of AIBN (a radical initiator), the oxygen reacts initially with the alkyl radicals of fatty acid chains in the triglyceride to form primary oxidation products—the hydroperoxides. 

Those highly reactive hydroperoxides follow several pathways, leading to oxygenated and cross-linked products. For example, they react preferably with the allylic hydrogens to form relatively stable allyl radicals. Those radicals in turn, can react with oxygen finally yielding some oxygenated products (e.g., epoxides, cyclic peroxides, aldehydes, alcohols) or they can recombine with other alkyl radicals yielding cross-linked glycerides. The ratio of the products attributable to propagation and termination steps is mostly influenced by the relative rates of branching and termination pathways [46]. 

In the IR and Raman spectra of the oxidized oil bands due to hydroxy group are absent. This supports the assumption that the reaction, does not stop at the hydroperoxide or epoxide stage. 

On the other hand, during such oxidative treatment radical polymerization of the vegetable oil occurs, resulting in the increase of viscosity. Examination of the ^1^H-NMR spectra reveals lack of peaks that can be attributed to epoxides [47]: 3.00 ppm for protons on the epoxy ring is missing and the peak at 1.50 ppm for the methylene protons adjacent to these epoxy groups shows only a negligible intensity. Signals that can be attributed to aldehydes and free acids are barely above the noise level. 

Combined evaluation of IR, Raman and NMR data supports the assumption that the double bonds were mainly converted to C-C bonds and ether (C-O-C) and/or peroxy (C-O-O-C) bridges.

## 3. Materials and Methods

### 3.1. Abyssinian Oil Modification

The oxidation processes of Abyssinian oil were conducted using equipment composed of three devices: a “limbo” pressure reactor (Büchi, Uster, Switzerland), a metering pump (Blue Shadow, Berlin, Germany) and a thermostat (Julabo, Seelbach, Germany). Starting materials crambe oil, 0.05% (*w*/*w*) NHPI and 0.05% (*w*/*w*) AIBN were fed to the pressure reactor. Then, the reactor was pressurized with 0.25 MPa with oxygen, the temperature was raised to 100 °C and the pressure was increased to 10 MPa with carbon dioxide. For reactions in pure oxygen, the reactor was pressurized with 0.4 MPa. During the process, the temperature and pressure were controlled. If necessary, pressure in the reactor was corrected by the addition of oxygen (for the process without solvent) or CO_2_ (for the process with solvent). 

### 3.2. Raman Spectroscopy

Raman spectra were obtained using an NRS 5100 confocal grating Raman microspectrometer (Jasco Corporation, Tokyo, Japan) which was equipped with a pumped laser with wave length of 532 nm and a Charged Coupled Device (CCD) detector. The operating conditions of the spectrometer were as follows: diffraction grating 1800 lines/mm, laser power 5.1 mW, numerical aperture 4000 μm, resolution 8.4 cm^−1^, lens magnification 20×, exposure time 15 s. 

The method of research was developed. The procedure was the same for each sample. Samples were applied to glass slides having a recess in order to maintain the same thickness of the sample layer. The measurement conditions were established (focusing on the sample, selection of integration time, number of scans, laser power). The Raman spectra of the oils were measured under selected conditions in a way to obtain the appropriate signal-to-noise ratio in the shortest possible time in the entire spectral range. The number of scans of the sample was 5, the exposure time was 15 s for a single accumulation, a laser with an excitation line of 532 nm was used and the measurements were taken at room temperature.

Obtained spectra were the basis for assessing the degree of unsaturation of the tested oils. An intensity ratio of bands at 1656 and 1444 cm^−1^ was taken into consideration for the analysis. The bands at 1266 and 1300 cm^−1^ in the analyzed oils were not very characteristic and they were not analyzed.

### 3.3. UV-VIS Spectroscopy

Spectra were recorded on a JASCO V-650 spectrometer (Jasco Corporation, Tokyo, Japan) using a 1 cm cuvette. The absorption spectra of the oils diluted with *n*-hexane (C = 5 × 10^−^^5^ g/mL) were measured in the region 200–400 nm. 

### 3.4. FT-IR Specrocsopy

FTIR-ATR spectra were recorded on Nicolet 6700 FT-IR spectrometer (Thermo Scientific, Waltham, MA, USA) equipped with a 60° ZnSe Attenuated Total Reflectance (ATR) accessory. The operating conditions of the spectrometer were as follows: wavelength range of 4000–650 cm^−1^, 32 scans, resolution of 4 cm^−1^. 

### 3.5. NMR Spectroscopy

^1^H-NMR spectra were recorded on Varian spectrometer (Palo Alto, CA, USA) at a frequency of 600 MHz using NMR solvent (CDCl_3_) which was purchased from ACROS Organics (Geel, Belgium). Coupling constants (*J*) are in Hertz (Hz). All chemical shifts (δ) are expressed in ppm downfield from TMS as an internal standard.

### 3.6. Determination of Modified Oils Properties

The viscosity-temperature oil properties were evaluated based on the kinematic viscosity determined at 40 °C and 100 °C in accordance with the PN EN ISO 3104:2004 norm and viscosity index in accordance with the PN-ISO 2909:2009 norm. The obtained modified oils were tested through the determination of kinematic viscosity and peroxide number in accordance with the PN-EN ISO 3960:2017-03 norm and iodine number in accordance with the PN-EN ISO 3961:2013-10 norm and saponification number in accordance with the PN-EN ISO 3657:2013-10 norm and acid number in accordance with the PN-EN ISO 660:2010 norm.

## 4. Conclusions

The aim of the research was to determine the changes in the chemical structure of oil components caused by its oxidation. For this purpose Raman, UV-Vis, IR and NMR spectroscopy were used for the sample analysis. In a way, all methods confirmed the structure changes occurring during the oxidative modification process. The analysis of the obtained Raman and NMR spectra led to the determination of the degree of fatty acids’ unsaturation. Furthermore, the change in the absorption bands and hence structural changes can be confirmed with the use of the IR and UV-Vis spectra. Spectroscopic methods are effective in observing the changes caused by the oxidation of *Crambe abyssinica* oil and determining the degree of unsaturation of fatty acids.

## 5. Patents

The research described above is the subject of two patent applications concerning vegetable oils’ modification entitled “Sposób Modyfikacji Olejów Roślinnych” by Iłowska, J.; Grabowski, R.; Szmatoła, M.; Gniady, J.; Woch, J.; Chrobak, J.; Korasiak, K.; Dejnega, B.; Szwach, I.; Fiszer, R. Patent Applications No. P.425863 and P.425863, which were submitted on 11 June 2018.

## Figures and Tables

**Table 1:** Analytical figures for the starting material (raw Abyssinian oil (A)) and the oil modified in the presence of the solvent (A_CO_2_) and in oxygen atmosphere, without solvent (A_O_2_).

Oil	A		A_CO_2_		A_O_2_	
	Mean Value	Standard Deviation	Mean Value	Standard Deviation	Mean Value	Standard Deviation
Kinematic viscosity at 40 °C, mm^2^/s	48.20	±0.35	143.40	±0.54	156.90	±0.26
Kinematic viscosity at 100 °C, mm^2^/s	10.10	±0.05	16.10	±0.07	17.20	±0.08
LN, meq O_2_/kg	2.80	±0.09	13.10	±0.31	15.16	±0.54
LJ, g I_2_/100 g	83.48	±0.65	63.70	±0.57	54.10	±0.48
LZ, mg KOH/g	170.50	±0.76	248.70	±0.91	214.23	±0.67
LK, mg KOH/g	0.34	±0.04	28.05	±0.04	25.70	±0.02

Conditions: reaction time 5 h; temperature 120 °C; NHPI 0.05% (*w*/*w*); AIBN 0.05% (*w*/*w*); stirring at 1000 rpm, oxygen pressure 2.5 MPa, total pressure 10 MPa (A_CO_2_); oxygen pressure maintained at constant 0.4 MPa (A, A_O_2_).

**Table 2:** Properties of the starting materials and results of the PetroOxy tests.

Oil	A		A_CO_2_		A_O_2_	
	Mean Value	Standard Deviation	Mean Value	Standard Deviation	Mean Value	Standard Deviation
VG viscosity class, ISO 3448	46	-	150	-	150	-
LJ, g I2/100 g	83.48	±0.65	63.70	±0.57	54.10	±0.48
Oxidation induction time at 80 °C, h	32.80	±2.62	2.71	±0.22	2.38	±0.19
Oxidation induction time at 120 °C, h	2.55	±0.20	0.66	±0.05	0.67	±0.05

**Table 3:** Chemical shifts and vibrational modes present in the Raman spectra of oils.

Raman Shift, cm^−1^	Bond	Group	Vibrational Mode
3100–2800	=C-H, C-H	-CH_3_, -CH_2_	stretching
1656	C=C	*cis* RCH=CHR	stretching
1444	C-H	-CH_2_	stretching
1300	−C-H	-CH_2_	scissoring
1266	=C-H	-CH_2_	twisting
1087	C-C	-(CH_2_)_n_	stretching

**Table 4:** Chemical shifts and integral values in ^1^H-NMR spectra of crude oil A and oils after oxidation (A_CO_2_ in the presence of CO_2_ and A_O_2_ without CO_2_).

Proton	δ [ppm]	Integral		
		*Crambe abyssinica* Oil	A_O_2_	A_CO_2_
CH_2_CH**_3_**	0.88	7.08	7.81	7.75
(CH**_2_**)_n_	1.35–1.20	60.23	62.36	62.13
CH**_2_**CH**_3_**	1.65–1.55	5.96	7.38	7.09
CH**_2_**-CH=CH-CH**_2_**-	2.10–1.97	9.37	6.94	7.21
CH**_2_**COO	2.34–2.28	5.34	6.12	6.08
CH=CH-CH**_2_**-CH=CH	2.83–2.75	1.34	0.35	0.38
OCH**_2_**CH(O)CH**_2_**O	4.32–4.12	3.65	3.83	3.74
OCH_2_CH(O)CH_2_O	5.28–5.20	0.94	0.93	0.88
CH=CH	5.43–5.28	5.54	3.42	3.58
